# A robust reprogramming strategy for generating hepatocyte-like cells usable in pharmaco-toxicological studies

**DOI:** 10.1186/s13287-023-03311-w

**Published:** 2023-04-18

**Authors:** Guillem Garcia-Llorens, Teresa Martínez-Sena, Eugenia Pareja, Laia Tolosa, José V. Castell, Roque Bort

**Affiliations:** 1 Unidad de Hepatología Experimental y Trasplante Hepático, Instituto de Investigación Sanitaria La Fe, Hospital Universitario y Politecnico La Fe, Torre A. Lab 6.08, Avda. Fernando Abril Martorell 106, 46026 Valencia, Spain; 2 Centro de Investigación Biomédica en Red de Enfermedades Hepáticas y Digestivas (CIBERehd), Instituto de Salud Carlos III, Madrid, Spain; 3 Departamento de Bioquímica y Biología Molecular, Universidad de Valencia, Valencia, Spain; 4 Servicio de Cirugía General y Aparato Digestivo, Hospital Universitario Dr. Peset, Valencia, Spain; 5 Centro de Investigación Biomédica en Red de Bioingenieria, Biomateriales y Nanomedicina (CIBER-Bbn), Instituto de Salud Carlos III, Madrid, Spain

**Keywords:** Hepatocyte-like cells, Direct reprogramming, Hepatocyte, Pharmaco-toxicology, Inborn errors of metabolism, iHEP, HLC

## Abstract

**Background:**

High-throughput pharmaco-toxicological testing frequently relies on the use of established liver-derived cell lines, such as HepG2 cells. However, these cells often display limited hepatic phenotype and features of neoplastic transformation that may bias the interpretation of the results. Alternate models based on primary cultures or differentiated pluripotent stem cells are costly to handle and difficult to implement in high-throughput screening platforms. Thus, cells without malignant traits, optimal differentiation pattern, producible in large and homogeneous amounts and with patient-specific phenotypes would be desirable.

**Methods:**

We have designed and implemented a novel and robust approach to obtain hepatocytes from individuals by direct reprogramming, which is based on a combination of a single doxycycline-inducible polycistronic vector system expressing HNF4A, HNF1A and FOXA3, introduced in human fibroblasts previously transduced with human telomerase reverse transcriptase (hTERT). These cells can be maintained in fibroblast culture media, under standard cell culture conditions.

**Results:**

Clonal hTERT-transduced human fibroblast cell lines can be expanded at least to 110 population doublings without signs of transformation or senescence. They can be easily differentiated at any cell passage number to hepatocyte-like cells with the simple addition of doxycycline to culture media. Acquisition of a hepatocyte phenotype is achieved in just 10 days and requires a simple and non-expensive cell culture media and standard 2D culture conditions. Hepatocytes reprogrammed from low and high passage hTERT-transduced fibroblasts display very similar transcriptomic profiles, biotransformation activities and show analogous pattern behavior in toxicometabolomic studies. Results indicate that this cell model outperforms HepG2 in toxicological screening. The procedure also allows generation of hepatocyte-like cells from patients with given pathological phenotypes. In fact, we succeeded in generating hepatocyte-like cells from a patient with alpha-1 antitrypsin deficiency, which recapitulated accumulation of intracellular alpha-1 antitrypsin polymers and deregulation of unfolded protein response and inflammatory networks.

**Conclusion:**

Our strategy allows the generation of an unlimited source of clonal, homogeneous, non-transformed induced hepatocyte-like cells, capable of performing typical hepatic functions and suitable for pharmaco-toxicological high-throughput testing. Moreover, as far as hepatocyte-like cells derived from fibroblasts isolated from patients suffering hepatic dysfunctions, retain the disease traits, as demonstrated for alpha-1-antitrypsin deficiency, this strategy can be applied to the study of other cases of anomalous hepatocyte functionality.

**Supplementary Information:**

The online version contains supplementary material available at 10.1186/s13287-023-03311-w.

## Introduction

Current liver in vitro models for drug screening and pharmaco-toxicological testing rely on hepatic-lineage cells cultured under standard culture settings. Gold-standard cell model is primary cultured human hepatocytes, but they are scarce and expensive for routine testing. On the other hand, the hepatoma cell line HepG2 is robust, cheap and easy to handle but performs a limited number of hepatocyte functions (reviewed in [1]) and display features of neoplastic transformation. Other cellular models exist, such as HepaRG [2] or Upcyte hepatocytes [3], that perform better than HepG2 but are rather expensive and more labor demanding when being used.

In the last few decades, extensive efforts have been made to develop strategies to produce human hepatocyte-like cells (HLC) by direct lineage conversion (reviewed in [4]) and pluripotent stem cells (PSC) differentiation (reviewed in [5]). HLC generated through direct reprogramming or PSC differentiation from the same donor displayed similar functionality and equally improved their phenotype after in vivo repopulation [6]. Although HLC do not yet display a fully mature phenotype, they outperform many other liver cell models and the degree of differentiation achieved is generally sufficient for most study goals. These cells undergo profound metabolic changes toward the hepatic phenotype and in fact, we have recently published how HLC suffer a complete rewiring of glutamine/glutamate metabolism mimicking that of actual hepatocytes [7]. Generation of human HLC by direct lineage conversion has been achieved by different strategies (recently reviewed in [4]). This is usually accomplished by ectopic expression of key liver enriched transcription factors, most frequently HNF4A and either FOXA1, FOXA2 or FOXA3. We selected expression of HNF4A, FOXA3 together with HNF1A based on previous studies [8] and the robustness of this combination in our own laboratory [7, 9–11]. However, since differentiation stops cell growth, generation of large quantities of HLC requires an additional step to override the proliferation arrest induced by the expression of these factors. This can be achieved by the expression of oncogenes such as MYC [12–14] or large-T antigen [8, 9] sometimes combined with silencing of tumor suppressor p53 [12, 14]. However, all these strategies suffer from an important shortcoming in pharmaco-toxicological testing, i.e., malignant traits caused by oncogene expression occur in these HLC [15] that may distort the significance of the results. Implementation of cellular in vitro models in pharmaco-toxicological testing also requires reproducibility, reliability, low cost and simplicity [16]. All these features are necessary and desirable to successfully implement a cellular model in high-throughput highly automated platforms used in testing thousands of chemicals. Strategies based on the use of PSC-derived HLC are technically demanding and use expensive growth factors [17, 18].

In this study, we have analyzed different approaches to obtain HLC by direct conversion of human dermal fibroblasts by exogenous expression of HNF4A, HNF1A and FOXA3 using either a) independent constitutive lentiviral vectors (HLC-3F), b) a doxycycline-inducible polycistronic lentiviral vector (TetO-HHFG; diHLC), c) TetO-HHFG expression in Large-T antigen immortalized HDF (diHLC-LT) and d) TetO-HHFG expression in hTERT-transduced HDF (diHLC-T). We selected the fourth strategy (diHLC-T) since it can deliver HLC from cultured cells even after at least 110 population doublings (reproducibility). Expansion and reprogramming protocols use commercial, easy-available and affordable cellular media (low cost) and it is achieved under standard cell culture conditions in 10 days (simplicity). The generated HLC are usable for pharmaco-toxicological studies.

## Methods

### Plasmids and lentivirus generation

The lentiviral expression vector pLV-hTERT-IRES-hygro [19] and FUW-M2rtTA [20] were obtained from Addgene (#85140 and #20342, respectively). The lentiviral vector TetO-HHFG (Additional file 1: Fig. S1A) was described in detail previously ([9]. Lentivirus was generated in 293 T cells by cotransfection of pHIV vector with pPAX2 and pMD2.G in 10:7.5:5 ratio. Lentivirus was collected and concentrated using Lenti-X concentrator following manufacturer´s instructions (Takara). We routinely performed two analysis to confirm lentivirus production and concentration. First, we used Lenti-X GoStrix (Takara) to confirm a titer above 5 × 10^5^ infectious units per ml after concentration. Second, we always produced in parallel a control lentiviral vector expressing GFP (pHIV-eGFP-FOXA3) and infect human fibroblasts. GFP-positivity should be higher than 75% by flow cytometer.

### Cell culture procedures

Wild-type (wt) human dermal fibroblasts (HDF) were purchased from ATCC® (CRL-2429). Disease HDF were isolated from a punch skin biopsy from homozygous alpha-1-antitrypsin deficiency (AATD) with Pi*ZZ genotype patient. HDF were isolated as described in detail in http://www.bu.edu/dbin/stemcells/protocols.php. Briefly, skin biopsy was incubated overnight at 37 °C in 1 mL digestion media (DMEM high glucose containing 20% fetal bovine serum, 23,500 U. Collagenase type I, 20 mg DNAse I and 1% penicillin–streptomycin). Next day, digested skin was vortexed for 20 s., centrifuged at 1500 g × 3 min and resuspended in 4 mL of incubation media (DMEM high glucose containing 20% fetal bovine serum and 1% penicillin–streptomycin). Cells were seeded in a T25 flask and left untouched for 3 days.

We expanded the life span of HDF by expression of human telomerase reverse transcriptase (hTERT; HDF-T). For this purpose, HDF were infected with the lentivirus vector pLV-hTERT-IRES-hygro. Forty-eight hours later, 400 μg/ml hygromycin was included in cell media. Surviving cells were expanded in 400 μg/ml hygromycin and stocked.

**Fig. 1:**
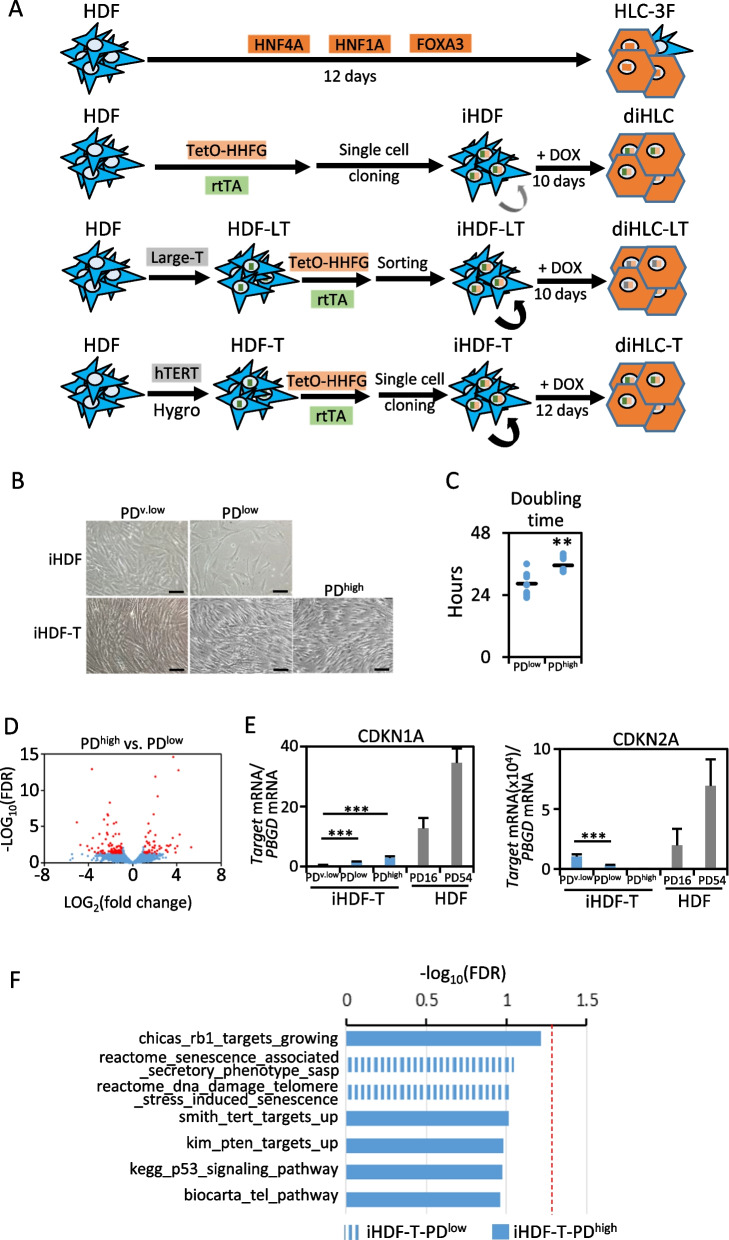
hTERT-transduced cell lines are phenotypically stable without senescence traits. **A** Schematic representation of the different strategies employed to obtain hepatocyte-like cells (HLC) from human dermal fibroblasts (HDF), i.e., exogenous constitutive expression of HNF4A, HNF1A and FOXA3 using independent lentiviral vectors (HLC-3F), a doxycycline-inducible polycistronic lentiviral vector expressing HNF4A, HNF1A and FOXA3 (TetO-HHFG; diHLC), TetO-HHFG expression in large-T antigen immortalized HDF (diHLC-LT) and TetO-HHFG expression in hTERT-transduced HDF (diHLC-T). **B** Representative phase contrast images of iHDF and iHDF-T at PD^v.low^ (just derived; approximately PD24), PD^low^ (PD 30–40) and PD^high^ (PD 100–110). Images depict confluent cultures when possible. Black bar equals 100 μm. **C** Estimated doubling time for PD^low^ and PD^high^ iHDF-T (*n* = 6; three replicates each). **D** Volcano plot of all genes expressed in PD^high^ vs. PD^low^ iHDF-T (three replicates each) by RNAseq. Plotted genes were expressed in all samples of at least one group (absolute reads > 10). Red dots correspond to genes differentially expressed defined as FDR < 0.05 and fold change > 2 (0.6%). **E** mRNA level of *CDKN1A* and *CDKN2A* in iHDF-T at PD^v.low^, PD^low^ and PD^high^. Gray bars represent primary human dermal fibroblasts at PD16 and PD54 (early senescent). Values correspond to the average of 2 experiments with four replicates each. ***p < 0.005. **F**None of the senescence-related gene sets was significantly different between PD^low^ and PD^high^ iHDF-T according to GSEA (see details in the text). Filled bars: NES > 0; Dashed bars: NES < 0. Red line marks FDR = 0.05

Clonal inducible iHDF and iHDF-T (Fig. 1A) were obtained by infecting HDF or HDF-T with 1:1 mixture of reprogramming lentivirus generated with TetO-HHFG and FUW-M2rtTA. The fraction of infected cells was ≈40% as determined in a separate well by GFP-positivity after a 24-h pulse of 1 µg/mL of doxycycline (Dox). Cells were then cloned by dilution cloning as described [21]. Briefly, six days after co-infection cells were trypsinized and resuspended in DMEM high glucose containing 20% fetal bovine serum and 1% penicillin/streptomycin. A suspension of 3 cells per mL was obtained by 1/100 serial dilution. Two hundred microliter cell suspension per well was seeded in 96-well plates (0.5–1 cell/well). Plates were kept at 37 °C and 5% CO_2_ for 2 weeks with a media change after 1 week. Wells containing cells were expanded and GFP-positive clones, after a 48-h Dox pulse, stocked in liquid nitrogen. GFP-positivity was re-tested after thawing. iHDF-LT derivation was described previously [7]. All types of HDF and iHDF cells were cultured in DMEM containing 10% fetal calf serum.

Dox-induced hepatocyte-like cells, i.e., diHLC, diHLC-LT and diHLC-T (Fig. 1A), were obtained by culturing iHDF, iHDF-LT and iHDF-T, respectively, in hepatocyte maturation media (HMM) [8] containing 250 ng/mL Dox for 12 days. Epidermal growth factor (EGF) and transforming growth factor beta (TGFβ) were purchased to PeproTech. In parallel, fibroblasts were maintained in the same media and conditions without Dox as control plates (iHDF-T_HMM_). Media was changed every other day. All media was purchased from ThermoFisher. Cells were maintained at 37 °C with 5% CO_2_ and were regularly examined with an Olympus CKX41 microscope. Cell proliferation was assessed as previously described [22].

HepG2 cells (ECACC No.85011430) were seeded as previously described [23]. For toxicometabolomic and high content screening (HCS) analysis, cultured cells (HepG2 and diHLC-T) were incubated on 12-well plates by triplicated with several referenced drugs, amiodarone, azathioprine, diclofenac, acetaminophen, bupropion, cyclophosphamide and stavudine at 1, 10, 100 and 1000 μM for 20 h. Samples defined as blanks are wells incubated with culture medium in the absence of cells. Meanwhile, the control samples are wells incubated under the same conditions but without toxic compound.

Human liver samples were obtained from three donors.

Human hepatocytes were isolated from liver biopsies (< 5 g), using a two-step collagenase perfusion technique [24]. Hepatocytes were seeded on fibronectin/collagen-type I-coated (Sigma-Aldrich, Madrid, Spain) dishes and cultured with Ham’s F-12/Williams (1:1) medium (Gibco BRL, Paisley, Scotland). Seeded hepatocytes were allowed to settle for 6 h before experiments were initiated.

### HCS assay: incubation of fluorescent probes, imaging and analysis

Following amiodarone incubation for 24 h, cells were simultaneously loaded with several fluorescent dyes to measure multiple biomarkers of cell toxicity (mitochondrial injury, ROS production and viability). Different combinations of fluorescent probes were used to identify specific mechanisms of toxicity according to previously described HCS assays [25]. Briefly, Hoechst 33,342 nucleic acid stain was used for sensitive and accurate cell number determination, PI exclusion for cell viability and exclusion of death cells in HCS analysis, TMRM for mitochondrial membrane potential and CellROX dye as an indicator of oxidative stress damage. After incubating with dyes, cells were imaged by the IN Cell Analyzer (GE Healthcare). Dyes were excited, and their fluorescence was monitored at the excitation and emission wavelengths at appropriate filter settings. The collected images were analyzed using the IN Cell workstation analysis module, which allows the simultaneous quantification of subcellular structures that are stained by different fluorescent probes. The measured fluorescence intensity was associated with the predefined nuclear and cytoplasmic compartments. Briefly, the nucleus was defined as the main object using an edge detection algorithm. Cytoplasm was defined using a “region growing” and “threshold” algorithm. In order to separate individual cells, segmentation was applied. Cell viability was determined by propidium iodide exclusion in the main object. Oxidative stress induction was detected CellROX fluorescence intensity in the cytoplasm. Cellular MMP was defined as the TMRM fluorescence intensity in punctuate cytosolic regions around the nucleus. Data is expressed as percentage of solvent-treated cells.

### RT-qPCR, immunofluorescence, periodic acid-Schiff (PAS) staining, ELISA and evaluation of CYP450s activities

RT-qPCR, immunofluorescence and PAS staining were performed as previously described [15]. Control Liver RNA sample was obtained by combining human liver total RNA (ThermoFisher) from three donors. Primer sequences are shown in Additional file 2: Table S1. RT-qPCR expression was determined by the 2^∆Ct^ using PBGD gene (HMBS) as normalizer. Primary antibodies were as follows: Albumin (Bethyl Lab. A-80-229A; 1/500), alpha-1-antitrypsin (Cell Marque 223A-15; 1/500) and Ki67 (ThermoFisher R-9106; 1/500). Alexa488-Phalloidin was acquired from LifeTech and used at 200U/mL). Fluorescence images were taken in a Leica DM2500 microscope. Quantification of immunofluorescence results was performed using CellProfiler software [26].

To determine the presence of human albumin and alpha-1 antitrypsin in cell media, we used ELISA Quantitation Set kits (Bethyl Laboratory) according to the manufacturer´s instructions.

To assess biotransformation activity, a cocktail containing substrates of the major CYP450 enzymes was incubated during 24 h. Production of phase I metabolites was monitored in cell media by liquid chromatography–tandem mass spectrometry (HPLC–MS/MS) as described [27].

### RNAseq analysis

Total RNA was isolated using MicroElute Total RNA Kit (Omega Bio-Tek R6831). Bioanalyzer’s RQN values were at least 9.9 for all RNAs. The mRNA in total RNA was converted into a library of template molecules of known strand origin using Illumina® TruSeq® Stranded mRNA LT Sample Prep Kit. Sequencing was performed in an Illumina platform following the manufacturer’s instructions. Total reads per sample were between 32 and 78 million.

RNA expression levels were extracted from the RNAseq data using featureCounts function available in Bioconductor R package Rsubread [28], after alignment on GRCh38 with STAR [29] of trimmed reads by Trim Galore!. (http://www.bioinformatics.babraham.ac.uk/projects/trim_galore/). Previously, these trimmed reads had been processed to remove ribosomal RNA contamination sequences using Burrows–Wheeler Aligner [30]. After mapping, the data was filtered keeping only in the analysis those genes which had base mean cutoff value > 10 reads. Bioconductor DESeq2 package [31] for the R software was used for further analysis. In particular, two-group differential expression analysis was performed using Deseq package; raw p values were adjusted using the Benjamini–Hochberg procedure and a false discovery rate (FDR) cutoff of 0.05 in the analysis was used as statistical significant threshold. Alternatively, unsupervised analysis was performed by variance stabilizing transformation which includes log transformation. Transformed data was filtered for non-informative values [32]. Finally, hierarchical clustering, heatmap and principal component analysis (PCA) were performed in Metaboanalyst (www.metaboanalyst.com).

### Gene ontology (GO) and gene set enrichment analysis (GSEA)

GO analysis was performed in Gene Ontology AmiGO (https://amigo.geneontology.org/amigo) [33] using the Bonferroni correction for multiple testing and a FDR < 0.05. For pathway analysis, normalized data from RNAseq was filtered for duplicate symbols and analyzed using GSEA software (Broad Institute) according to published methods [34]. Briefly, data was overlapped on selected gene sets downloaded from the Molecular Signature Database and measured for the enrichment of genes at the top or bottom of the gene list to determine their correlation with the gene set's phenotype. The GSEA parameters used included: metric for ranking genes, signal2noise; enrichment statistic, weighted; permutation type, gene_set. Gene sets significantly enriched were identified using a multiple hypothesis testing FDR < 0.05. When evaluating a specific pathway that includes upregulated and downregulated gene sets, for example, KRAS.300_UP.V1_UP and KRAS.300_UP.V1_DN, only those with NES > 0 and NES < 0, respectively, were considered.

### Metabolomic sample preparation

Cell monolayers were cleaned twice with cold PBS and scratched with 300 µL of a cold solution of methanol/water (3:1 v/v) with internal standards (phenylalanine-D5, tryptophan-D5 and caffeine-D9) at 0.25 μM. Supernatant was collected and evaporated to dryness under vacuum and reconstituted in 75 μL of 95% water and 5% acetonitrile with 0.1% (v/v) formic acid. A volume of 25 μL of each sample was pooled jointly to prepare the quality control (QC) sample. Protein quantification was performed by Lowry assay. Azathioprine 1000 µM samples had protein content lower than 3-times blank samples content and were removed from further analysis.

### Liquid chromatography high-resolution mass spectrometry

Analysis was performed using an Agilent 1290 Infinity UPLC chromatography coupled to an iFunnel quadrupole time of flight (QTOF) Agilent 6550 spectrometer (Agilent Technologies, CA, USA). Samples were analyzed by a Synergi Hydro-RP C18 (150 × 1 mm, 4 µm) column (Phenomenex, Torrance, USA) that was heated to 50 °C in the column oven. The mobile phases consisted of acetonitrile with 0.1% (v/v) formic acid as solvent B and water with 0.1% (v/v) formic acid as solvent A. Sample analysis was initially performed by 1% of solvent B for 2 min. Then, a linear gradient of solvent B from 1 to 80% on 8 min was observed. Finally, 98% of solvent B was held for 2 min and then initial conditions for 3 min to allow reconditioning of the system. The flow rate used was 0.4 mL/min over 15 min chromatogram total run time. Electrospray positive mode was used for these experiments. To correct mass drifts during the acquisition, a reference standard of phthalic anhydride, purine and hexakis (1H, 1H, 3H-tetrafluoropropoxy) phosphazine was used. The samples are injected randomly and QC sample was injected every eight samples to correct instrumental drift.

### Metabolomic data processing and software

For intrabatch correction, a nonparametric QC-SVRC approach was used as described elsewhere [35]. We removed features that were not at least sixfold higher than the mean detected in blank samples. Seventy-one and sixty-four signals were successfully identified using MS–MS data, while 228 and 274 metabolites were identified by MS level for diHLC-T and HepG2, respectively, using the Human Metabolome Database (www.hmdb.ca) and METLIN (www.metlin.scripps.edu) with 20 ppm accuracy.

Analysis of mass spectrometry data required a raw data conversion to open data performed by proteowizard software [36]. Preprocessing of data for untargeted analyte profile including peak detection, deconvolution, alignment and integration was performed by XCMS v 3.4.2 [37] software in R v 3.5.0. Statistical analysis was carried out in MATLAB 2021a (Mathworks Inc., Natick, MA, USA). Metabolic pathways of metabolites are defined by the KEGG database (www.genome.jp).

### Other data analysis

All the data are expressed as mean ± SD values and represent triplicate measurements of independent experiments. For statistical analysis, real values of control and test compounds were compared. A Student’s t test was used for the statistical evaluations calculated with GraphPad Prism vs. 6.1. The chosen significance level was p < 0.05.

## Results

### hTERT bypass senescence in human dermal fibroblasts (HDF) cell lines inducible to hepatocyte-like cells (HLC) without neoplastic transformation

We recently reported the generation of an inducible immortal human fibroblast cell line (iHDF-LT) capable to differentiate to hepatocyte-like cells upon doxycycline (Dox) addition (diHLC-LT; Fig. 1A) [9]. This cell line combines the expression of large-T antigen with a polycistronic vector containing HNF4A, HNF1A and FOXA3 cDNA controlled by seven tandem copies of a tetracycline responsive element (TetO-HHFG, Additional file 1: Fig. S1A). diHLC-LT displayed hepatocyte morphology and acquired several basic hepatocyte functions. To preliminary assess hepatocyte conversion, we evaluated the expression of liver-specific *ALB* mRNA. The expression was sixfold lower than HLC generated by independent expression of HNF4A, HNF1A and FOXA3 (HLC-3F, Fig. 1A, Additional file 1: Fig. S1B). To avoid the deleterious effect of large-T antigen expression in HLC functionality [8], we designed a fibroblast cell line containing TetO-HHFG without the use of large-T antigen (iHDF) (Fig. 1A). For this purpose, we delivered a 1:1 mix of lentiviral vectors TetO-HHFG and rtTA (reverse tetracycline-controlled transactivator) to commercial HDF at passage 4. After single-cell cloning, 15 clones grew and 5 of them were above 90% GFP-positive after a 48-h Dox pulse (i.e., Dox responsive). We calculated population doubling 24 (PD24) for the cloned iHDF cells frozen in these original vials containing approximately 15 × 10^6^ cells (≈2^24^). After reprogramming with Dox, average expression of *ALB* mRNA in Dox-induced hepatocyte-like cells (diHLC) was apparently lower than HLC-3F, although it did not reach statistical significance (Additional file 1: Fig. S1B). Single-cell cloned iHDF entered senescence and stop proliferating after eight additional population doublings (Fig. 1B), limiting our experimental setting and overall utility of the model. In conclusion, implementation of polycistronic inducible vectors in iHEP reprogramming requires a fibroblast cell line resistant to senescence without neoplastic transformation.

As an alternative to expression of oncogenes such as large-T antigen or Myc, we used hTERT to expand the life span of human dermal fibroblasts. Commercial HDF at passage 4 were infected with a lentiviral vector constitutively expressing hTERT (Fig. 1A; diHLC-T). After hygromycin selection, we obtained a pooled population of resistant fibroblasts expressing hTERT (HDF-T). HDF-T were then infected with a 1:1 mix of lentiviral vectors TetO-HHFG and rtTA and single-cell cloned as described above. We seeded 240 wells rendering a total of 57 grown clones and 13 of them were above 90% GFP-positive after a 48-h Dox pulse (i.e., Dox responsive). Cells were expanded and Dox response (GFP-expression as surrogate marker) re-validated after thawing. Three clones (P3G4, P3C7 and P5E10) were re-tested over time and 98, 93 and 90% of the cells were GFP-positive after Dox pulse and less than 1% positive without Dox (Additional file 1: Fig. S2). We calculated PD24 for the clonal inducible fibroblasts expressing hTERT (iHDF-T) contained in these original vials (PD^v.low^). When cultured in HMM containing 250 ng/ml Dox for 10 days, doxycycline induced hepatocyte-like cells (diHLC-T) acquired an epithelial morphology and the induction of selected hepatocyte mRNA, except TAT was above that of diHLC-LT (Additional file 1: Fig. S1C). A similar scenario was found when compared to diHLC, expression of selected liver markers, except ALB and HGD, was several fold induced (Additional file 1: Fig. S1D). mRNA level for senescence markers *CDKN1A* and *CDKN2A* was several fold lower than in diHLC with similar PD (Additional file 1: Fig. S1E).

Next, we expanded and stocked three clones until PD30-40 (PD^low^) and one clone (P3G4) until PD110-120 (PD^high^) using a splitting ratio of 1:10 every week. We did not detect any morphological evidence of senescence (Fig. 1B). PD^low^ and PD^high^ iHDF-T grew similarly in culture with doubling times of 28.4 ± 4.4 h and 35.5 ± 3.0 h, respectively (Fig. 1C). To get a deeper insight into the phenotypic differences between PD^high^ and PD^low^ iHDF-T, we performed expression profiling by RNA sequencing (RNAseq). The overall gene expression pattern was strikingly similar between both cell populations (GSE204867), of more than 20,000 mapped genes, only 139 (0.6%) were differentially expressed (FDR < 0.05; twofold) (Fig. 1D, Additional file 2: Table S2). GO enrichment analysis did not result in any significantly enriched pathway (FDR < 0.05) in any of the three subclasses (i.e., biological process, molecular function and cellular component).

Minor changes in specific cellular pathways might be overseen in a global expression profile analysis. Thus, we focused on detail in the acquisition of senescence features in PD^high^ iHDF-T. *CDKN1A* mRNA slowly increased with cell passes but remained far below the levels present in low passage HDF cells (PD16); *CDKN2A* mRNA decreased with passages and was hardly detectable at PD^high^ (Fig. 1E). To point out any modest but coordinate change in the expression of genes functionally related to senescence, we performed gene set enrichment analysis (GSEA). MSigDB_C2 gene sets with the wild card search “senescen*” and GO0048144 (GO term fibroblast proliferation) were not significantly enriched (FDR < 0.05) (Fig. 1F). We conclude that iHDF-T cells do not accumulate senescent features after a long cell-passing protocol.

**Fig. 2:**
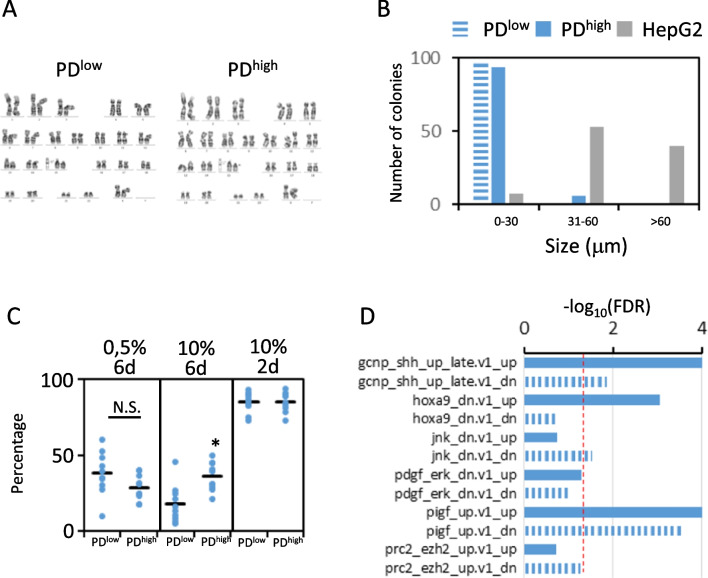
hTERT-transduced cell lines do not acquire neoplastic features at PD^high^. **A** iHDF-T karyotype is not altered after more than 70 population doublings: 46XX,del (15) (q11.2q15). Twenty-five cells per cell population were analyzed. **B** iHDF-T do not grow under soft matrix compared to carcinogenic HepG2 cells. A total of 100 cell aggregations per cell type were analyzed. **C** Percentage of Ki67-positive cells in cultures of PD^low^ and PD^high^ iHDF-T. Cells were cultured 6 days at 0.5% serum, 6 days at 10% serum and 2 days at 10% serum. A total of 10 images from cultured cells immunostained with anti-Ki67 antibody were analyzed using ImageJ software. Number of cells counted was 360, 470 and 300 for 6d-0.5%; 6d-10% and 2d-10%, respectively. * p < 0.05 **D** Most significant oncogenic molecular signatures in PD^high^ vs. PD^low^ iHDF-T (GSEA). Filled bars: NES > 0, enriched in PD^high^. Filled bars: NES < 0, enriched in PD^low^. Red line marks FDR = 0.05. Note that HOXA9, JNK and PDGF-ERK gene sets contain genes up and downregulated after silencing of the procarcinogenic genes in malignant cell lines

The life span of human fibroblasts can be significantly expanded by hTERT expression [38]. To confirm the absence of cancer-associated changes, we analyzed karyotype instability, growth in soft support and loss of contact inhibition or serum-independent proliferation in PD^low^ and PD^high^ iHDF-T. Karyotype analysis confirmed euploidy and genomic stability of iHDF-T after 80 population doublings (Fig. 2A). Similarly, no anchorage independent growth was found in PD^low^ or PD^high^ iHDF-T (Fig. 2B), a characteristic of normal human fibroblasts. When grown for 2 days in complete media in low confluence, approximately 85% of cells were Ki67-positive (Fig. 2C). However, when cultured with low serum, cells did not reach confluence after 6 days, and the percentage of Ki67-positive cells drop to approximately 30% (37.9 ± 14.2 and 28.4 ± 8.5 for PD^low^ and PD^high^, respectively, not significant, *n* = 9). Moreover, the percentage of Ki67-positive cells was not higher in PD^high^ iHDF-T, excluding a progressive decline in the requirement of exogenous growth factors to proliferate, a typical feature of cellular transformation. Finally, Ki67-positivity dropped when grown at confluence and we did not detect cells piling up. Nevertheless, Ki67-positivity was higher in PD^high^ cells (35.9 ± 9.3 vs. 17.7 ± 12.4, p < 0.005). GSEA using all datasets contained in C6 (oncogenic signatures; size: 30–500; *n* = 190) resulted in minimal enrichment in non-biologically relevant gene sets (Fig. 2D).

We, therefore, conclude that iHDF-T display the desirable features of being cells with an extended life span (> 110 doublings), not showing neoplastic transformation traits and reprogrammable to hepatocyte-like cells under basic culture conditions.

### iHDF-T are a reliable and robust cell system for the generation of homogeneous hepatocyte-like cells at large scale

Once the cellular properties of iHDF-T were defined, we directed our interest into the phenotype of diHLC-T, in particular to stocks with high population doublings (PD110-PD120). Before addressing the phenotypic analysis, we tested Dox dose–response, incubation time, cell density and media renewal rate in our experimental setting regarding hepatic induction (Additional file 1: Fig. S3). Based on our results, we selected doxycycline 250 ng/ml for 12 days, with media change every other day and seeding of 40,000 cells per cm^2^ 24 h before Dox incorporation.

**Fig. 3:**
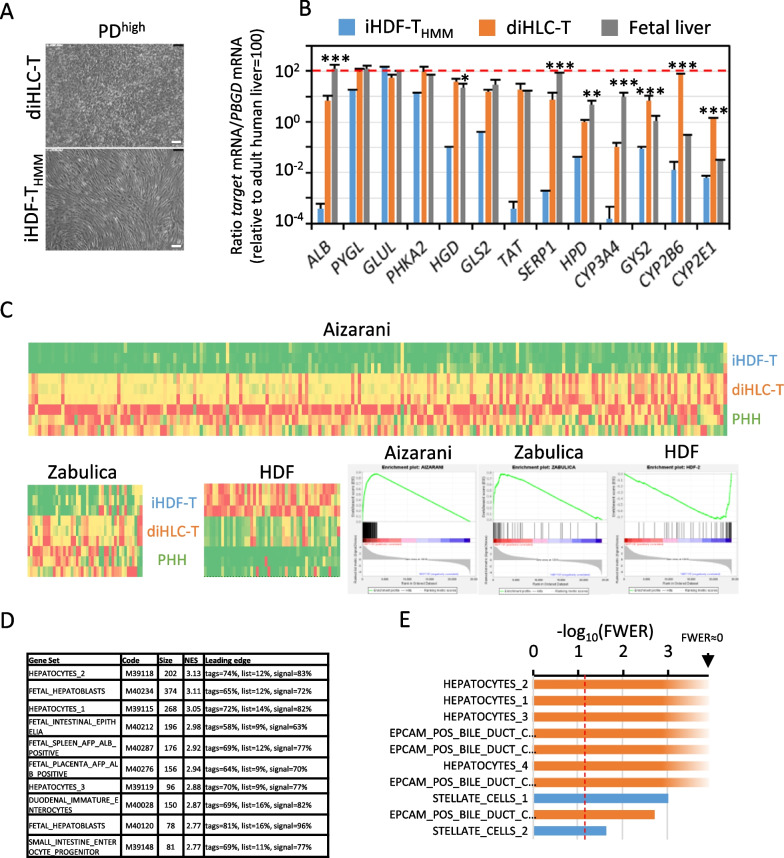
PD^high^ diHLC-T gene expression resemble human hepatocytes. iHDF-T were reprogrammed to diHLC-T by incubation in HMM media containing 250 ng/mL Dox for 12 days. Control cells were cultured in parallel without Dox (iHDF-T_HMM_). **A** Representative phase contrast images of iHDF-T_HMM_ and diHLC-T after 12 days culture. White bar equals 100 μm. **B** mRNA levels of multiple hepatic gens in diHLC-T. Values correspond to the average plus standard deviation of two different experiments (four samples each) determined by RT-qPCR and expressed relative to adult human liver (pooled RNA from 3 donors). Fetal liver values were obtained by comparison with values extracted from GSE15238 [60]. Statistical significance between diHLC-T and fetal liver is included.*p < 0.05; **p < 0.01; ***p < 0.005. **C** RNAseq extracted heatmaps depicting expression of genes within Aizarani_hepatocyte single-cell [39], Zabulica [40] and archetypal HDF genes (Additional file 2: Table S3). Enrichment plots (GSEA) are shown on the right. **D** Table containing the most significant (FDR > 0.05) signature gene sets determined by GSEA using C8 MSigDB cell types signature gene sets (https://www.gsea-msigdb.org/gsea/msigdb/genesets.jsp?collection=C8). **E** Bar graph depicting the most significant (FWER) signature gene sets determined by GSEA using all liver 31 single-cell signature gene sets reported by Aizarani [39]. Orange: NES > 0, enriched in diHLC-T. Blue: NES < 0, enriched in iHDF-T. Red line marks FDR = 0.05. As expected, stellate cells, a quiescent fibroblast cell present in the liver, correlate with iHDF-T

Dox addition triggered a mesenchymal to epithelial transition (Fig. 3A). We measured by RT-qPCR the mRNA levels of selected hepatocyte gene markers comprising immature/embryonic (*ALB, PYGL, GLUL, PHKA2, HGD, GLS2, TAT* and *SERPINA1*) and mature/adult hepatocytes (*HPD, CYP3A4, GYS2, CYP2B6* and *CYP2E1*). According to this limited analysis, we considered our cells between immature and mature hepatocytes under standard cell culture conditions (Fig. 3B). We extended this transcriptional analysis to all genes by RNAseq of PD^high^ iHDF-T and diHLC-T. Heatmap and GSEA analysis using Aizarani_hepatocyte [39] and Zabulica gene sets [40] as well as an archetypal HDF gene markers (Additional file 2: Table S3) confirmed silencing of fibroblast gene network and commitment to the hepatocyte gene expression profile (Fig. 3C). The hepatocyte-like identity of diHLC-T was confirmed by GSEA analysis over MSigDB_C8 gene sets (cell type signature) (Fig. 3D). Finally, GSEA analysis using 31 gene set clusters based on single-cell RNAseq in the human liver [39] confirmed the hybrid embryonic/mature phenotype of our diHLC-T cells (Fig. 3E). Note the significant correlation of HDF with stellate cells (blue bars), which are fibroblast-like cells present in the liver.

**Fig. 4:**
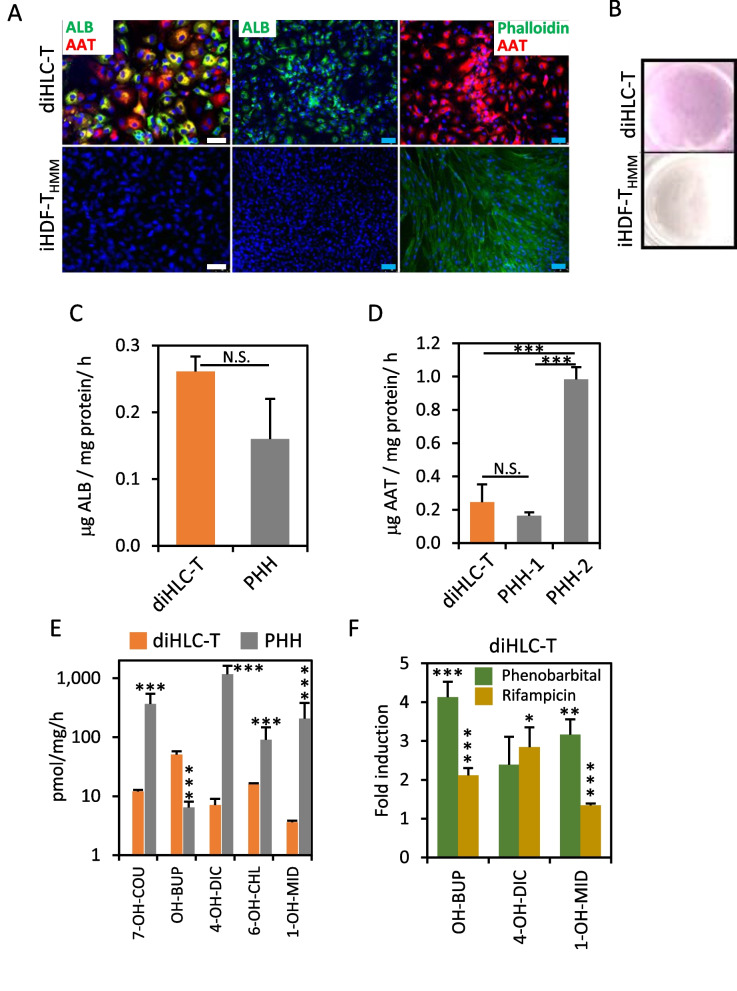
PD^high^ diHLC-T perform hepatocyte functions. **A** Representative fluorescence images of cells immunostained with antibodies against human albumin and α1-antitrypsin. Actin filaments were visualized by incubation with Alexa Fluor™ 488 Phalloidin. Nuclei were stained with DAPI. White bar equals 50 μm. Blue bar equals 100 μm. **B** Accumulation of glycogen determined by periodic acid-Schiff staining. **C** Human albumin secretion was estimated from 24-h cell media using an ELISA kit. PHH: cultured primary human hepatocytes from two different donors. **D** α1-antytrypsin secretion was estimated from 24-h cell media using an ELISA kit. PHH: cultured primary human hepatocytes. All values correspond to average plus standard deviation. **E** Relevant CYP450 activities compared to PHH. The metabolic products of coumarin (7-hydroxycoumarin, assay for CYP2A6 activities), bupropion (hydroxybupropion, assay for CYP2B6 activity), diclofenac (4-hydroxydiclofenac, assay for CYP2C9 activity), chlorzoxazone (6-hydroxychlorzoxazone, assay for CYP2E1 activity) and midazolam (1-hydroxymidazolam, assay for CYP3A4 activity) were determined by liquid chromatography–tandem mass spectrometry. Values correspond to the average of at least two experiments with three replicates each. *p < 0.05; **p < 0.01; ***p < 0.005. **F** Induction of CYP450 activities by phenobarbital and rifampicin in diHLC-T. Activities were quantified as in (**E**). Values correspond to the average of two experiments with three replicates each. Statistical significance for fold induction was obtained by comparison with solvent-treated cells.*p < 0.05; **p < 0.01; ***p < 0.005

Hepatocyte functionality of diHLC-T beyond gene expression was confirmed at different levels. Our cells expressed high levels of albumin and alpha-1 antitrypsin (Fig. 4A) and stored glycogen in the cytoplasm (Fig. 4B). They secreted albumin and alpha-1 antitrypsin within the range of human hepatocytes (Fig. 4C, D). We detected significant levels of relevant CYP450 monooxygenases (Fig. 4E). Moreover, these activities were induced under canonical CYP450 chemical inducers, i.e., phenobarbital and rifampicin (Fig. 4F).

**Fig. 5:**
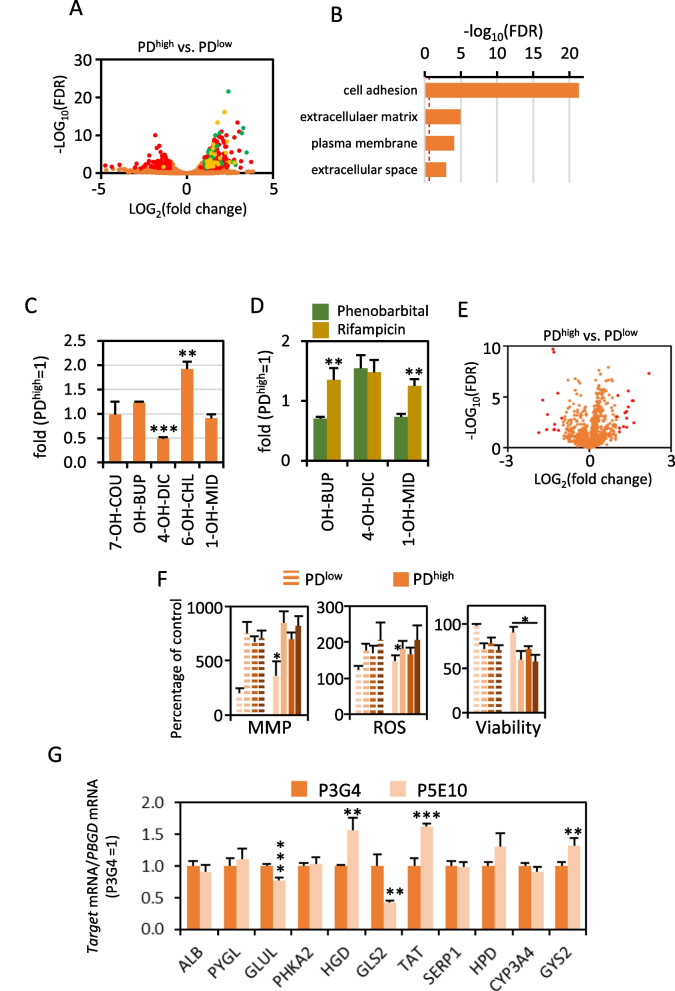
Robust induction of the hepatocyte program in iHDF-T. iHDF-T maintain inducibility after more than 80 population doubling without losing hepatic phenotype in diHLC-T. **A** Volcano plot of all genes expressed in PD^low^ and PD^high^ diHLC-T (three replicates each) by RNAseq. Genes with higher expression in PD^high^ diHLC-T are depicted as LOG_2_(FC) > 0. Plotted genes were expressed in all samples of at least one group (absolute reads > 10). Red, green and yellow dots correspond to genes differentially expressed defined as FDR < 0.05 and fold change > 2. Green dots represent genes encoding hepatocyte-secreted plasma proteins. Yellow dots represent genes encoding hepatocyte-relevant secreted glycoproteins such as decorin (DCN) or fibromodulin (FMOD). A complete list of differentially expressed genes is included in Additional file 2: Table S4. **B** GO molecular function enrichment within differentially expressed genes. Dashed red line marks FDR = 0.05. **C** Comparison of relevant CYP450 activities between PD^low^ and PD^high^ diHLC-T. Activities were quantified as in Fig. 4E. **D** Induction of CYP450 activities by phenobarbital and rifampicin. Activities were quantified as in Fig. 4E. **E** Volcano plot of metabolomics features (m/z-Rt) quantified in PD^low^ and PD^high^ diHLC-T (six replicates each) by liquid chromatography-TOF. Features with LOG_2_(FC) > 0 are more abundant in PD^high^ diHLC-T. Plotted features were expressed in all samples of at least one group. Red dots correspond to features differentially abundant defined as FDR < 0.05 and fold change > 2. **F** HCS analysis of amiodarone incubation at 1–10-100–1000 µM in PD^low^ and PD^high^ diHLC-T (six replicates each). *p < 0.05. **G** Relative mRNA level of multiple hepatic genes in P5E10 clone (PD30-40). Values correspond to the average plus standard deviation of two different experiments (four samples each) determined by RT-qPCR and expressed relative to PD^low^ (P3G4). For comparison, relative mRNA levels of PD^low^ (PD30-40), PD.^high^ (PD100-110) and PD125 of P3G4 clone are depicted in Additional file 1: Fig. S4

We evaluated intra-clonal variability of our cell pharmaco-toxicological system model by RNAseq of PD^low^ and PD^high^ diHLC-T. For this purpose, PD^low^ and PD^high^ iHDF-T cells were reprogrammed with Dox. Similar to iHDF-T previously described, the overall gene expression pattern was highly similar between both diHLC-T populations (GSE204867) and of more than 20,000 mapped genes, only 363 (1.6%) were differentially expressed (FDR < 0.05; twofold; Fig. 5A; Additional file 2: Table S4). GO enrichment analysis indicated significant enrichment in extracellular matrix and hepatocyte-secreted plasma proteins in PD^high^ diHLC-T (Fig. 5A, B). Nevertheless, no difference was found in Aizarani or Zabulica gene sets (data not shown) or archetypal hepatic functions such as CYP450 activity and inducibility (less than a twofold difference; Fig. 5C, D). To further validate the homogeneity of diHLC-T after more than 80 passes, we performed an untargeted intracellular metabolomic profile. Of a total of 1139 features (m/z-Rt), only 20 (1.8%) were differentially abundant between PD^low^ and PD^high^ diHLC-T (Fig. 5E). Next, we compared the toxicity of amiodarone (1–1000 µM), a well-characterized hepatotoxicant [25, 41], in both diHLC-T by HCS using a multiparametric assay (Fig. 5F). Mitochondrial membrane potential and reactive oxygen alteration was equivalent in most concentrations except at the lowest compound concentration, 1 µM, where PD^high^ was more sensitive. Viability was slightly different (less than 20%) between both PD. In conclusion, we obtained a non-neoplastic fibroblast cell line, with a significant extension of their life span and capable to differentiate to hepatocyte after Dox addition without relevant alteration of its transcriptomic and metabolomics traits.

Finally, inter-clonal variability was also assessed by RT-qPCR of selected markers in two clones with similar population doubling. We found significant differences in 5/11 genes below the twofold range, thus with very limited biological relevance (Fig. 5G). For comparison, relative mRNA levels of PD^low^ (PD30-40), PD^high^ (PD100-110) and PD125 of P3G4 clone are depicted in Additional file 1: Fig. S4.

### diHLC-T are a robust and reliable cell culture model for toxicometabolomic studies

**Fig. 6:**
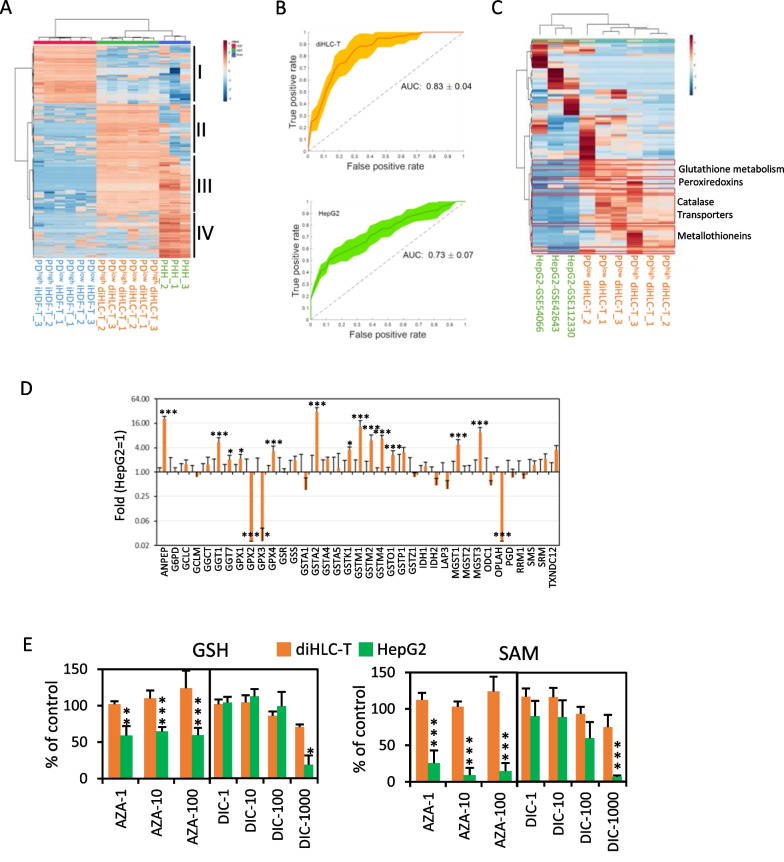
diHLC-T outperform HepG2 cells in toxicometabolomic testing. **A** Unsupervised hierarchical clustering of samples and genes obtained from RNAseq data (see details in methods). **B** ROC curve with 95% confidence interval (shadowed) of the 10-times repeated threefold CV for (top) diHLC-T and (bottom) HepG2 cells. **C** RNAseq extracted dendrogram/heatmap (sample and gene-wise) of genes present in DETOX gene set (GOBP: detoxification). Genes significantly enriched (*p* < 0.05) in diHLC-T are highlighted in a red box, while those genes significantly enriched (*p* < 0.05) in HepG2 are highlighted in a green box (only ABCB6, top). **D** mRNA levels of genes involved in GSH metabolism (KEGG: glutathione_metabolism). diHLC-T data is extracted from this study. HepG2 values are extracted from GSE42643[8], GSE54066[12] and GSE112330[14]. **E** Intracellular levels of reduced glutathione (GSH) and S-adenosyl-L-methionine (SAM) in diHLC-T and HepG2 cells. diHLC-T, *n* = 6; HepG2, *n* = 3; *p < 0.05, **p < 0.01,***p < 0.005)

Given the similarities between diHLC-T from very different cell population doublings, we performed hierarchical clustering of all classes: iHDF-T (PD^low^ and PD^high^), diHLC-T (PD^low^ and PD^high^) and primary cultured human hepatocytes (PHH). Dendrogram depicts a cluster composed of diHLC-T together with PHH (Fig. 6A). This clustering also evidences the similarities between cells from different PD. Four major gene clusters were formed (I–IV). Cluster I, highly expressed in iHDF-T, is enriched in extracellular matrix (GO:0031012, FDR = 3.89 × 10^–6^), cell adhesion (GO:0007155, FDR = 2.82 × 10^–15^) and protein binding (GO:0,005,515, FDR = 2.40 × 10^–3^). Cluster III, upregulated in diHLC and PHH, is enriched in all sort of metabolic pathways and activities. A full list of genes and GO terms are included in Additional file 3: Table S5A–E. In summary, diHLC-T have initiated the path toward human hepatocytes by silencing fibroblast transcriptome and inducing characteristic hepatocyte mRNAs.

Once determined the stability of diHLC-T with PD by HCS, we applied untargeted metabolomic analysis to establish their applicability for toxicometabolomic studies compared to HepG2, the most commonly used cell line in pharmaco-toxicological studies. A supervised multivariate partial least-squares discriminant analysis (PLS–DA) model was performed to identify differences among the metabolic profiles obtained for toxic compounds and controls (see details in Methods). To make the prediction models of toxicity, all the features identified by MS–MS and MS level were included in HepG2 and the reprogrammed models. Most of the identified metabolites belong to the glutathione metabolic pathways (glutathione, pyroglutamic, oxoproline…), arginine and proline metabolism (putrescine, spermine, creatinine…) and glycine, serine and threonine metabolism (choline, betaine, cysteine…) (Additional file 1: Fig. S5). diHLC-T model detected a greater number of metabolite changes in the major metabolic pathways altered between toxic versus non-toxic samples, as compared to HepG2 (Additional file 4: Table S6). The power of discrimination among classes (toxic vs. non-toxic) by each cell type was evaluated by ROC curves with all identified metabolites on both MS–MS and MS level. diHLC-T-based model showed an area under the ROC Curve (AUC) mean value above 0.8 while HepG2 cells model AUC mean value = 0.73 (Fig. 6B). A null generation of AUC values performed by a permutation test (*n* = 200) were carried out for both PLS-DA model showing a *p* < 0.05 on diHLC-T and HepG2 models. The ROC curve of the toxicity model of diHLC-T had a larger AUC (*p* < 0.05) suggesting better sensitivity and specificity of the toxicity model. Significant differences between mRNA levels for DETOX mRNA (GOBP: detoxification) and particularly glutathione metabolism (KEGG: glutathione metabolism) might be at the root of this improvement (Fig. 6C). Only ABCB6, a member of the ATP-binding cassette (ABC) transporter superfamily and related to drug resistance, out of 103 genes included in the database was significantly upregulated in HepG2 cells compared to diHLC-T. In fact, we found a significant increase in diHLC-T mRNA encoding enzymes participating in reduced glutathione transfer (Fig. 6D). The values of reduced glutathione (GSH) and S-Adenosyl-L-methionine (SAM) remained higher in diHLC-T, even at high concentrations of azathioprine and diclofenac (Fig. 6E).

### diHLC-T derived from a AATD patient recapitulate the altered phenotype

**Fig. 7:**
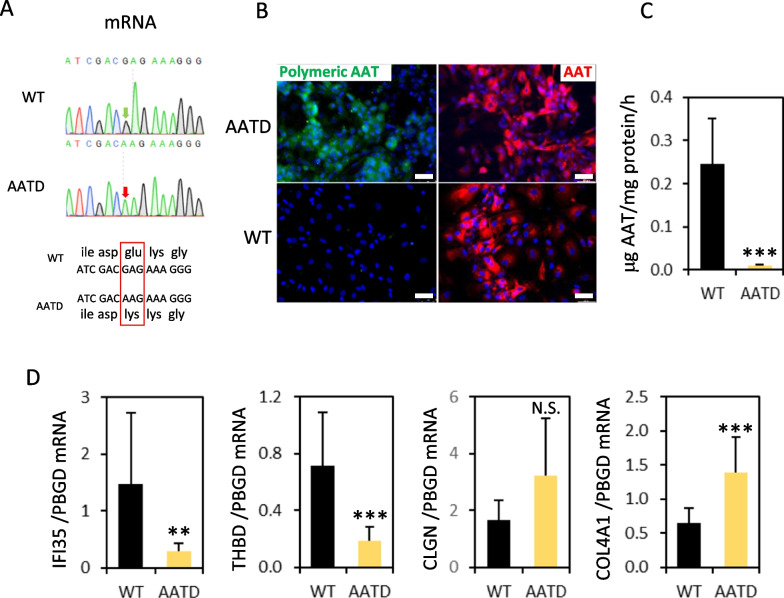
Characterization of disease phenotype in diHLC-T^AATD^. iHDF-T^AATD^ were reprogrammed to diHLC- T^AATD^ by incubation in HMM media containing 250 ng/ml DOX for 12 days. **A** Sequence of retrotranscribed *SERPINA1* mRNA isolated from diHLC-T^AATD^ corresponding to the region of exon 6 containing the Glu366Lys mutation (PiZZ genotype). **B** Representative fluorescence images of cells immunostained with antibodies against polymeric and standard human alpha-1 antitrypsin in diHLC-T and diHLC-T^AATD^. Nuclei were stained with DAPI. White bar equals 75 μm. **C** AAT secretion was estimated from 24-h cell media using an ELISA kit. Data correspond to the average of three different clones per genotype plus standard deviation. **D** mRNA level of four differentially expressed genes based on results previously published [42]. Values depicted correspond to the average of three different diHLC-T clones with WT and PiZZ genotype. All values correspond to average plus standard deviation

An important additional advantage of diHLC-T is the possibility to derive hepatocyte-like cells with specific liver diseases. We isolated HDF from a patient with AATD. Following the protocol depicted in Fig. 1, we obtained diHLC-T^AATD^. Dox addition induced a drastic change in morphology manifested by lose of elongated morphology and acquisition of a compact polygonal cell mass (Additional file 1: Fig. S6A). We then analyzed the mRNA expression of 10 genes as a surrogate markers of the hepatocyte phenotype including genes encoding enzymes of tyrosine metabolism (*TAT*, *HPD* and *HGD*), enzymes involved in glutamine/glutamate metabolism (*GLS2* and *GLUL*), enzymes involved in glycogen metabolism (*GYS2* and *PYGL*) and enzymes involved in xenobiotic metabolism (*CYP2B6*, *CYP2E1* and *CYP3A4*). According to our analysis, diHLC-T^AATD^ are rather immature hepatocytes in terms of mRNA expression (Additional file 1: Fig. S6B). Finally, expression of albumin and alpha-1 antitrypsin was detected in iHEP-T by immunofluorescence (Additional file 1: Fig. S6C). Of note, a drastic reorganization of actin filaments assembly was also noted in agreement with the mentioned cell shape change. *SERPINA1* mRNA was extracted from diHLC-T^AATD^ and sequenced after retrotranscription using specific primers spanning the PiZZ mutation. Reverse and forward sequencing of three clones revealed the same G > A substitution that results in a Glu366Lys amino acid change (Fig. 7A). Such amino acid modification causes the protein to polymerize. Polymerization of mutant alpha-1 antitrypsin impedes its secretion into cell media and accumulates in the endoplasmic reticulum. This could be demonstrated in reprogrammed cells using a monoclonal antibody specifically directed against the polymeric form specifically (Fig. 7B-C). The presence of the polymeric alpha-1 antitrypsin triggered the downregulation of inflammatory-related genes *IFI35* and *THBD* as well as upregulation of ER-associated gene COL4A1 (*CLGN* upregulation was not statistically significant) mRNA (Fig. 7D).

## Discussion

We have generated a fibroblast-derived cell line, with an extended life span beyond 110 doublings, without malignant traits and capable of being efficiently and simplistically reprogrammable into hepatocyte-like cells (HLC) under standard culture conditions. Reprogrammed cells perform multiple hepatocyte functions including those typical of mature hepatocytes such as CYP450 activity and inducibility. Gene set enrichment analysis and GOterm profile using data extracted from RNAseq classify the cells as mature/fetal hybrid hepatocytes. Toxicometabolomic analysis upon incubation with known active hepatic substances revealed that our diHLC-T outperform HepG2, probably due to the intense xenobiotic-related metabolic rewiring, including a general improvement of glutathione metabolic pathway. Moreover, the strategy envisaged here offers the possibility, neglected in HepG2 or HepaRG, to incorporate inherent human individual variability to in vitro modeling. In other words, this cellular reprogramming is a promising strategy for the development of in vitro models for drug safety/efficacy and disease modeling [10, 42].

It is worthy to underline that the generation of human HLC by direct lineage conversion results in a progressive cell division decay that ends in proliferation arrest [8, 12, 43]. Thus, generation of large quantities of HLC compatible with a widespread usage would require conferring proliferation capabilities, usually achieved by the expression of oncogenes. However, these strategies are accompanied by genomic instability, phenotypic changes, and altered growth requirements [44], which are not present when hTERT is used [45]. Thus, the presence of such malignant traits hampers the use of these cell in drug-induced oncogenicity and increases resistance to acute cytotoxicity [46–48]. To bypass such drawbacks, we successfully combined the expression of hTERT in the starting cell (human dermal fibroblasts) and the use of a polycistronic vector inducible by Dox.

Bypassing Hayflick proliferation limit by hTERT expression may pose a neoplastic burden. Some authors have shown that telomerase transduced cells can divide many passages without signs of neoplastic transformation [49–51]. While others have found the presence of neoplastic features such as chromosome anomalies at high population doublings or acquiring the ability to grow in the absence of solid support [52, 53]. Interestingly, our modified starting cells (iHDF-T) maintained a stable karyotype after 110 population doublings, did not form colonies in agar and retained the requirement of serum to proliferate and did not activate oncogenic pathways. As expected, PD^high^ iHDF-T expanded significantly their life span and did not acquire any senescence trait. Moreover, we confirmed the stability of iHDF-T features by global transcriptomic analysis of PD^high^ and PD^low^ iHDF-T.

We further confirmed the overall stability of reprogrammed diHLC-T by global transcriptional profiling and CYP450 functionality. Differential gene expression between PD^high^ and PD^low^ diHLC-T was limited to a small set of genes including secreted plasma proteins and hepatocyte-relevant secreted glycoproteins (higher expression at PD^high^). PCA clustered diHLC-T together without grouping by population doubling (Additional file 1: Fig. S7). Nevertheless, we still found some differences between PD^high^ and PD^low^ diHLC-T, in terms of gene expression, xenobiotic metabolism and toxicity, but those differences were less significant compared to differences found between cells from different donors [54]. Regardless of population doubling number, we define our diHLC-T as a hybrid immature/mature hepatocyte phenotype. Figure 3B shows that diHLC-T express, approximately, equal levels than fetal liver of most markers, but higher levels of GYS2, CYP2B6 or CYP2E1. Genes expressed at low levels in fetal vs. adult liver can be considered as mature markers (HPD, CYP3A4, GYS2, CYP2B6 and CYP2E1); thus, we concluded a hybrid immature/mature phenotype. Nevertheless, most CYP450 activities except CYP2B6/OH-BUP, depicted in Fig. 4E, are still significantly lower than in PHH and thus diHLC-T have not yet achieved a fully mature hepatocyte in the current culture conditions.

Hierarchical and k-means (three classes) clustering of samples clearly separated iHDF-T, diHLC-T and PHH. Clustering of genes confirmed an effective downregulation of fibroblast-specific genes in diHLC compared to iHDF-T. Among the genes upregulated in diHLC-T and highly present in PHH, we found those encoded by mtDNA, suggesting a higher content of mitochondria; in fact, hepatocytes are extremely rich in mitochondria [55].

Metabolomics based on liquid chromatography coupled to mass spectrometry can be successfully used to profile toxic response in vitro and in vivo [56, 57]. We have analyzed the cell extracts of HepG2 and diHLC-T incubated with reference hepatotoxicants to obtain a predictive model of toxicity caused by these drugs in both cell models. diHLC-T are able to maintain their normal metabolome at higher drug concentrations through expression of conjugation and other detoxification gene pathways. The results show that the predictive model based on the metabolome changes of diHLC-T cells has higher accuracy in classifying toxicity than HepG2.

Lowering the concentration of reduced glutathione (GSH) is acknowledged as an oxidative stress biomarker and is a key metabolite participating in conjugation reactions [58]. The fact that diHLC-T show higher GSH values explains their greater capability to protect themselves from oxidative damage better than HepG2. SAM is formed by methionine and ATP and is related to cell growth, differentiation and apoptosis. Decreased SAM values are reported as a consequence of liver injury [59]. Higher SAM values in diHLC-T may be due to maintained ATP values, as a more efficient preservation of mitochondrial function than HepG2 cells.

There is a need for in vitro models for pharmaco-toxicological testing that display inter-human variability or specific liver phenotypes. Current models used in toxicology, such as HepG2 or HepaRG, neglect such differences. For instance, they are not suitable in drug screening for hepatic inborn diseases such as glycogen storage or alpha-1 antitrypsin deficiencies. Such limitations can be overcome with proliferation-induced primary hepatocytes or iPSC-derived hepatocyte-like cells generated from such patients; still, it requires isolation and culture of patient hepatocytes which is ethically and economically challenging [3]. On the other hand, derivation of hepatocyte-like cells from induced pluripotent stem cells (iPSC) requires an additional step for iPSC derivation at the expense of a high economic burden, time and technological skills. The strategy here presented fulfills all the characteristics desirable for cells suitable for this type of toxicology studies and drug screening by its robustness, homogeneity and intrinsic capabilities for generating “disease” cell models, as illustrated in the case of AATD, reported here.

## Conclusion

The strategy envisaged in this report allows, at a reduced time and cost expenses, the generation of an unlimited source of clonal, homogeneous, non-transformed hepatocyte-like cells, capable of performing typical hepatic functions and suitable for pharmaco-toxicological high-throughput testing. Moreover, it could be applied in patient-derived cells for the study of hepatic diseases, such as inherited hepatic disorders and the screening of therapeutic drugs, as well as idiosyncratic drug-induced liver injury in given patients.

## Supplementary Information

**Additional file 1**. Supplementary Figures S1–S7.

**Additional file 2**. Supplementary Tables S1–S4.

**Additional file 3**. Supplementary Table S5.

**Additional file 4**. Supplementary Table S6.

## Data Availability

RNAseq data is available in GEO under code GSE204867 (https://www.ncbi.nlm.nih.gov/geo/). It includes raw “fastq.gz” files, extracted gene counts (not normalized) and metadata. Metabonomic data is available at zenodo.org with code 7761622. Additional data requests should be directed to the corresponding author.
