# Supplementary material for: A robust reprogramming strategy for generating hepatocyte-like cells usable in pharmaco-toxicological studies
Source: Stem Cell Res Ther. 2023 Apr 18;14:94. doi: 10.1186/s13287-023-03311-w (PMC10114490; doi:10.1186/s13287-023-03311-w)
Supplement: Supplementary file 1 — Additional file 1. Supplementary Figures S1–S7. [file 13287_2023_3311_MOESM1_ESM.pdf]

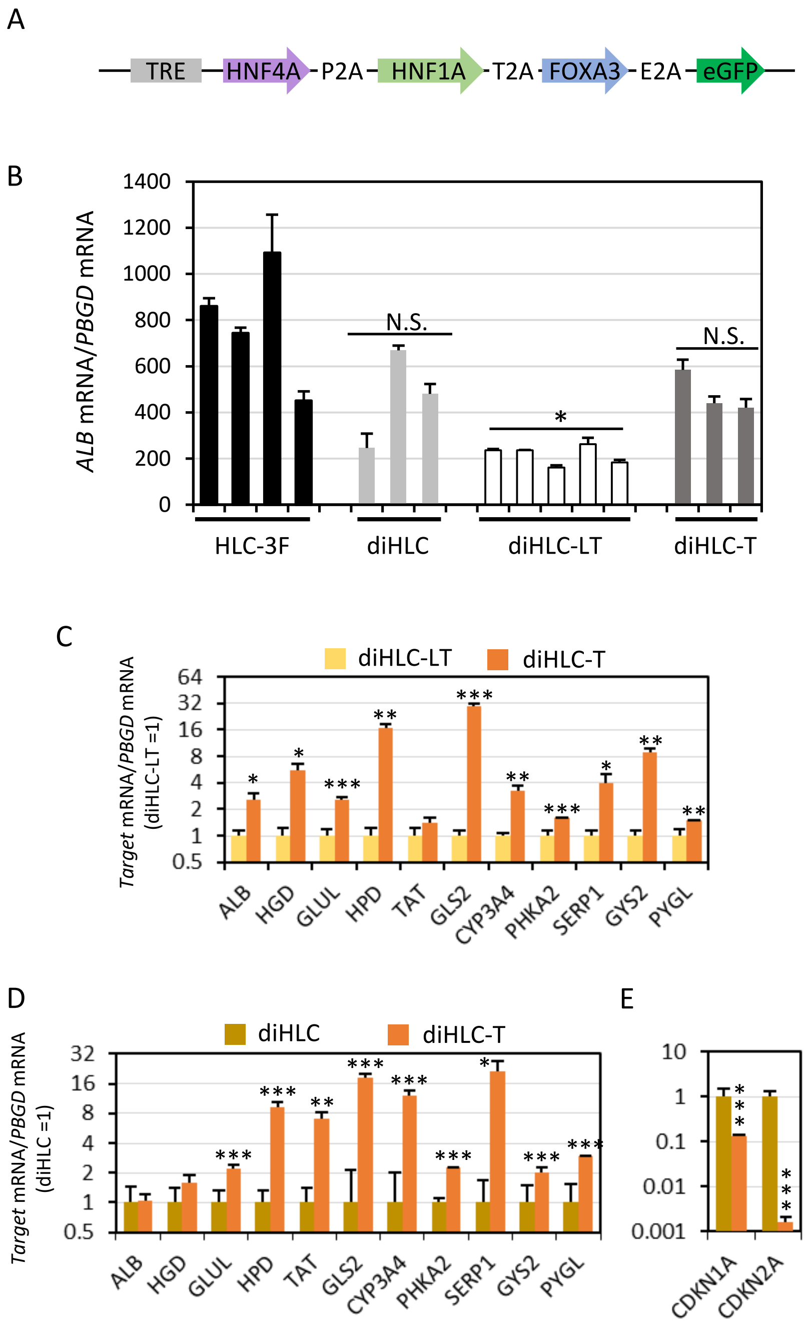

Figure S1. Direct hepatic reprogramming and mRNA level of selected hepatic genes. (A) Schematic representation of the TetO-HHFG expressing vector. TRE: 7x Tetracycline Responsive Element. **(B)** mRNA level of *ALB* in the different hepatocyte-like cell types. Each bar represents the average plus standard deviation of an independent experiment with 3 replicates each; * p<0.05 (comparison with HLC-3F). **(C)** Comparison between the mRNA level of selected hepatic genes between diHLC-LT and diHLC-T. Each bar represents the average plus standard deviation of 3-5 independent experiments; * p<0.05, ** p<0.01, *** p<0.005. **(D)** Comparison between the mRNA level of selected hepatic genes between diHLC and diHLC-T. Each bar represents the average plus standard deviation of 3-4 independent experiments; * p<0.05, ** p<0.01, *** p<0.005. **(E)** Relative *CDKN1A* and *CDKN2A* mRNA level in diHLC and diHLC-T at equal population doublings. Values were obtained by qRT-PCR from 2 and 3 independent experiments respectively (3-9 samples each).

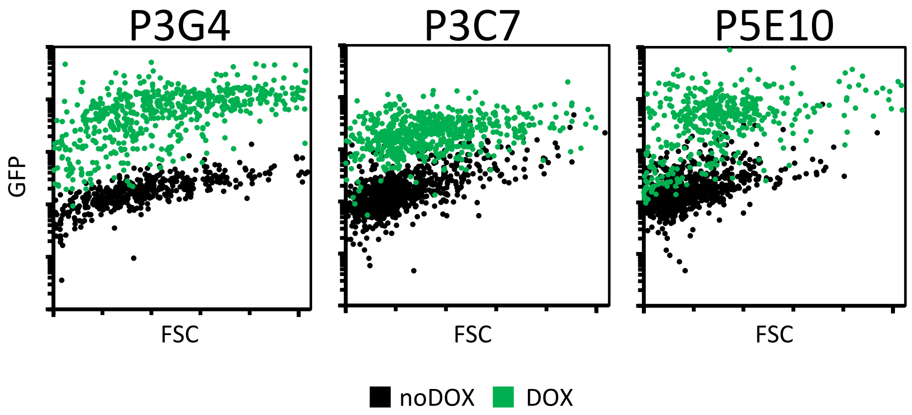

Figure S2. Expression of GFP (a surrogate marker for HNF4A, HNF1A and FOXA3) in iHDF-T with and without Dox. Cells were incubated in DMEM with 10% FCS and 250 ng/ml Dox for 48 hours. Three different clones, i.e. P3G4, P3C7 and P5E10, are depicted.

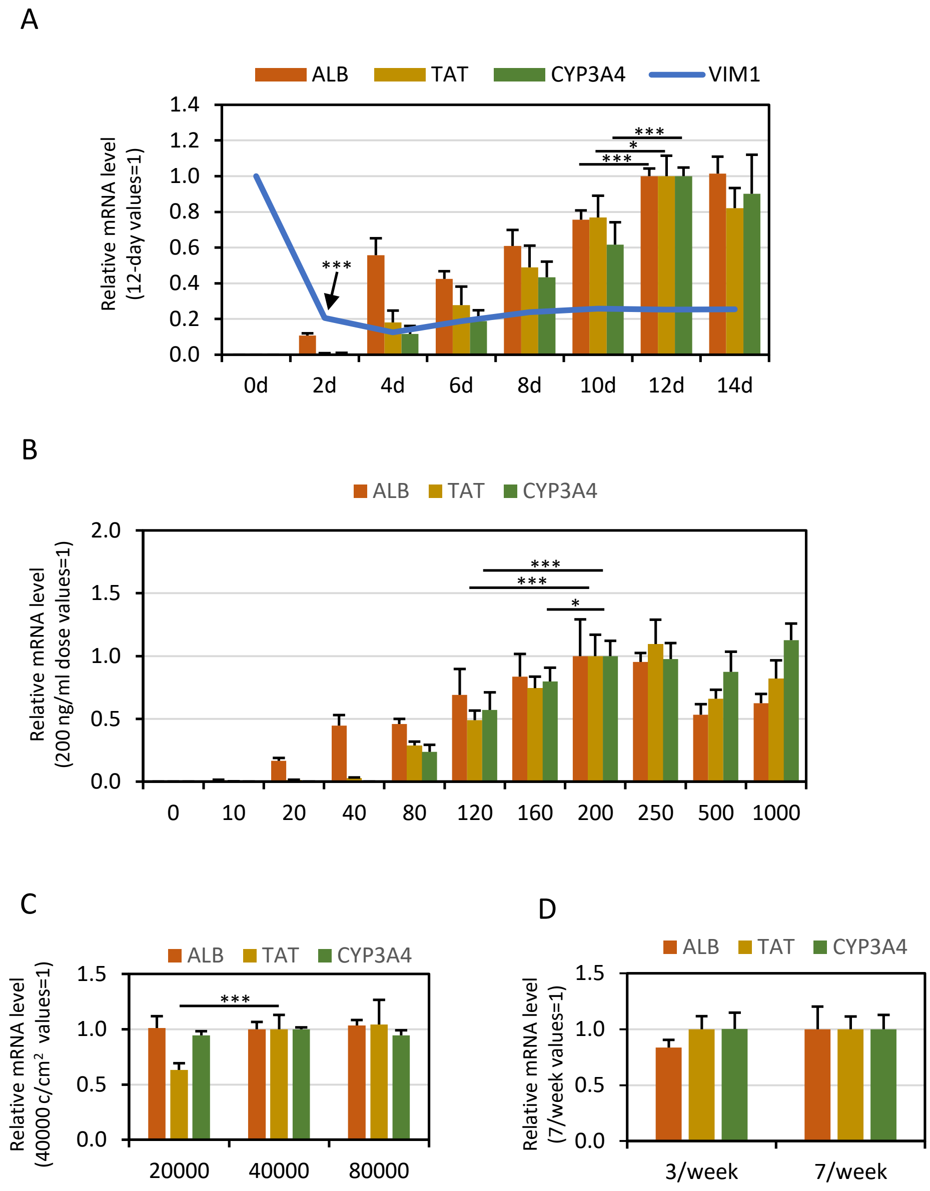

Figure S3. Defining culture conditions for diHLC-T reprogramming. iHDF-T were incubated in various conditions and the mRNA level of *ALB, TAT* and *CYP3A4* quantified by qRT-PCR and expressed relative to the maximum value. **(A)** Time curse from 0 to 14 days incubation in HMM media containing 250 ng/mL Dox. **(B)** Dose-response from 0 to 1000ng/ml of Dox. Cells were incubated for 14 days in HMM media. **(C)** Cells were seeded at 20000, 40000 and 80000 cells per cm^2, 24 hours before HMM media containing 250 ng/mL Dox was used. Cells were maintained for 12 days. **(D)** Cells were incubated in HMM media containing 250 ng/mL Dox for 12 days. Media was change either 3 times per week (Monday, Wednesday and Friday) or everyday (7/week).

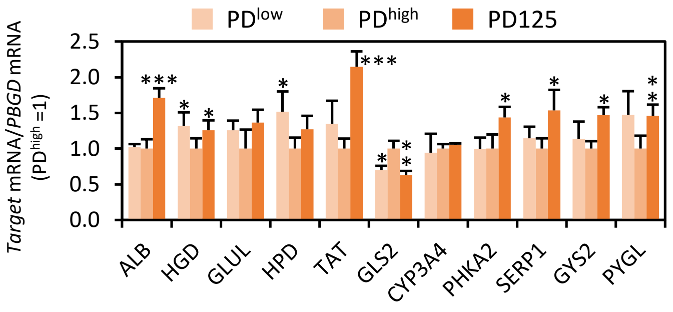

Figure S4. Similar expression of hepatic markers in PD^{low}, PD^{high} and PD$^{v.high}$ diHLC-T. iHDF-T from different PD were reprogrammed to diHLC-T by incubation in HMM media containing 250 ng/mL Dox for 12 days. Values correspond to the average plus standard deviation of 2 different experiments (4 samples each) determined by qRT-PCR and expressed relative to PD^{high} diHLC-T.

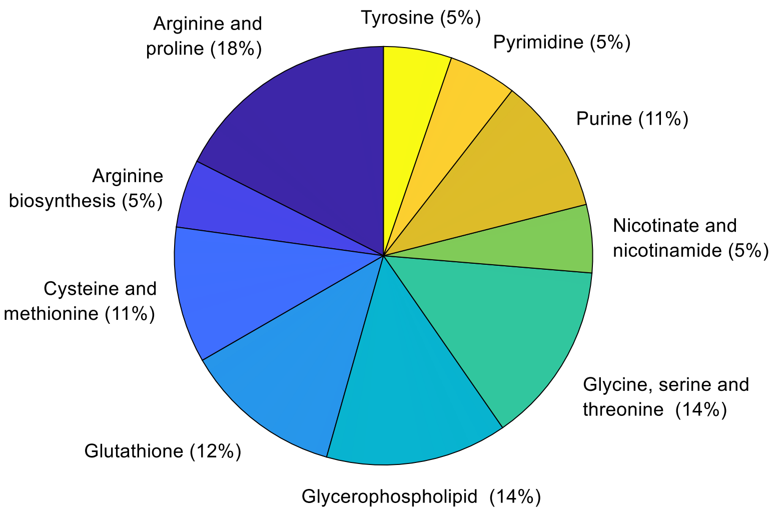

Figure S5. Main metabolic pathways with at least 3 metabolites identified in diHLC-T cells. Metabolic pathways are related to cell maintenance and viability such as the glutathione, glycerophospholipids, arginine and cysteine metabolism.

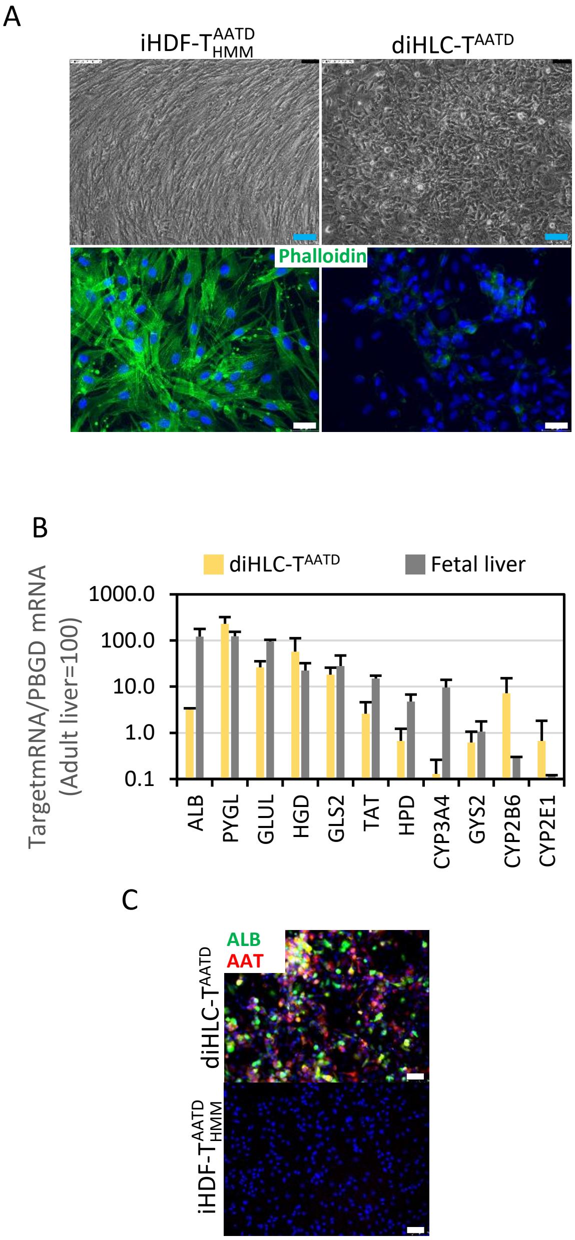

Figure S6. Reprogramming of iHDF-TAATD into hepatocyte-like cells. (A) Representative phase contrast and fluorescent images of iHDF-T^{AATD} cultured for 12 days in HMM without (iHDF-THMM) and with 250 ng/mL doxycycline (diHLC-T^{AATD}). Actin filaments were visualized by incubation with Alexa Fluor™ 488 Phalloidin. Nuclei were stained with DAPI. Blue and white bars equal 100 µm and 50 µm respectively. **(B)** mRNA level of multiple hepatic genes in diHLC-TAATD quantified by qRT-PCR. Values correspond to the average of 3 different clones (6 samples each) determined by qRT-PCR and expressed relative to adult human liver (pooled RNA from 3 donors). Fetal liver values were obtained by comparison with values extracted from GSE15238 (Tzur et al., 2009). **(C)** Representative fluorescence images of cells immunostained with antibodies against human albumin and alpha-1-antitrypsin. Nuclei were stained with DAPI. White and yellow bars equal 75 µm and 50 µm respectively.

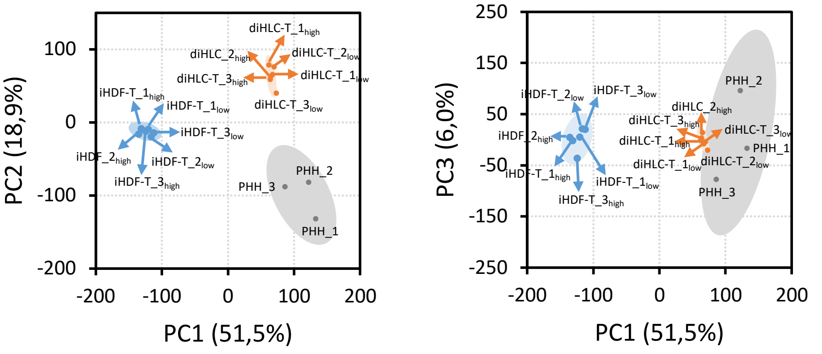

Figure S7. Principal Component Analysis of data depicted in Figure 6A.
